# Transcranial Static Magnetic Field Stimulation of the Motor Cortex in Children

**DOI:** 10.3389/fnins.2020.00464

**Published:** 2020-05-19

**Authors:** Asha Hollis, Ephrem Zewdie, Alberto Nettel-Aguirre, Alicia Hilderley, Hsing-Ching Kuo, Helen L. Carlson, Adam Kirton

**Affiliations:** ^1^Cumming School of Medicine, University of Calgary, Calgary, AB, Canada; ^2^Department of Pediatrics, Cumming School of Medicine, University of Calgary, Calgary, AB, Canada; ^3^Hotchkiss Brain Institute, Cumming School of Medicine, University of Calgary, Calgary, AB, Canada

**Keywords:** non-invasive brain stimulation, neurophysiology, pediatrics, neuromodulation, motor learning, tSMS

## Abstract

**Background:**

Non-invasive neuromodulation is an emerging therapy for children with early brain injury but is difficult to apply to preschoolers when windows of developmental plasticity are optimal. Transcranial static magnetic field stimulation (tSMS) decreases primary motor cortex (M1) excitability in adults but effects on the developing brain are unstudied.

**Objective/Hypothesis:**

We aimed to determine the effects of tSMS on cortical excitability and motor learning in healthy children. We hypothesized that tSMS over right M1 would reduce cortical excitability and inhibit contralateral motor learning.

**Methods:**

This randomized, sham-controlled, double-blinded, three-arm, cross-over trial enrolled 24 healthy children aged 10–18 years. Transcranial Magnetic Stimulation (TMS) assessed cortical excitability via motor-evoked potential (MEP) amplitude and paired pulse measures. Motor learning was assessed via the Purdue Pegboard Test (PPT). A tSMS magnet (677 Newtons) or sham was held over left or right M1 for 30 min while participants trained the non-dominant hand. A linear mixed effect model was used to examine intervention effects.

**Results:**

All 72 tSMS sessions were well tolerated without serious adverse effects. Neither cortical excitability as measured by MEPs nor paired-pulse intracortical neurophysiology was altered by tSMS. Possible behavioral effects included contralateral tSMS inhibiting early motor learning (*p* < 0.01) and ipsilateral tSMS facilitating later stages of motor learning (*p* < 0.01) in the trained non-dominant hand.

**Conclusion:**

tSMS is feasible in pediatric populations. Unlike adults, tSMS did not produce measurable changes in MEP amplitude. Possible effects of M1 tSMS on motor learning require further study. Our findings support further exploration of tSMS neuromodulation in young children with cerebral palsy.

## Introduction

Early brain injury can result in cerebral palsy (CP) and lifelong disability for millions (A Kirton, 2013a; Oskoui et al., 2016). Perinatal stroke (PS) is brain damage due to a focal disruption in cerebral blood flow occurring between 20 weeks gestation and 28 days postpartum (Dunbar and Kirton, 2018). PS causes most hemiparetic cerebral palsy (HCP) with disabling weakness on one side of the body. With no known treatment or prevention strategies, improving outcomes and quality of life in PS is focused on neurorehabilitation.

Non-invasive brain stimulation (NIBS) is an encouraging but understudied potential therapy for children with CP. Randomized trials suggest that repetitive transcranial magnetic stimulation (rTMS) (Gillick et al., 2015; Kirton et al., 2016) and transcranial direct current stimulation (tDCS) (Kirton et al., 2017; Gillick et al., 2018) may enhance motor learning in hemiparetic children. Proof-of-principle studies have demonstrated that the enhancement of motor learning seen in adults with tDCS of the primary motor cortex (M1) (Reis et al., 2009) also occurs in children (Ciechanski and Kirton, 2017; Cole et al., 2018). Although the safety of pediatric neurostimulation is becoming well established (Bikson et al., 2016; Friel et al., 2016), both rTMS and tDCS can have side effects, potentially limiting applications in younger children.

These neuromodulation approaches are based on evolving human and animal models of how the motor system develops following early unilateral injury (Eyre et al., 2007; Staudt, 2007a; Kirton, 2013b; Wen et al., 2018). Excessive preservation of motor control of the affected limb by the contralesional, ipsilateral hemisphere has led to trials trying “inhibitory” stimulation targeting contralesional M1. Animal models have also confirmed the optimal windows during which developmental motor plasticity occurs, with human equivalents occurring in infancy (Martin et al., 2011). Accordingly, a major limitation of existing neuromodulation approaches is difficulty of application in infants and toddlers, during the window in which one might expect the greatest potential therapeutic gains. There is therefore a need to find alternative forms of neuromodulation applicable at earlier stages of development.

Transcranial static magnetic field stimulation (tSMS) offers a potential solution. In tSMS, a strong magnet is held over the skull to generate a static magnetic field within functional cortical targets such as M1 (Oliviero et al., 2011; Kirimoto et al., 2016). Short-term application from 10 to 30 min in adults can decrease M1 excitability as assessed by the amplitude of transcranial magnetic stimulation (TMS)-generated motor-evoked potentials (MEPs). Original tSMS results have since been replicated (Silbert et al., 2013; Nojima et al., 2015; Nojima et al., 2016; Dileone et al., 2018) with only one study reporting no physiological changes (Kufner et al., 2017). tSMS effects have also been described in the cerebellum and parietal cortex (Carrasco-López et al., 2017; Matsugi and Okada, 2017). The effects of tSMS in the developing brain are unstudied.

Previous studies in the field have identified that use of south or north polarity did not alter the measured impact on cortical excitability, although most literature in the field still indicates use of south polarity by convention (Oliviero et al., 2011). Unlike polarity, magnet strength and duration of application are significant factors: stronger magnets (e.g. 45 × 30 mm versus 30 × 15 mm in size) and application for longer time periods (e.g. 30 min versus 10 min) have been shown to have a stronger and longer-lasting effect on cortical excitability.

Few investigations have explored the behavioral effects of tSMS. Two adult studies found that visual cortex tSMS could inhibit visual search performance and reduce experimental photophobia (Gonzalez-Rosa et al., 2015; Lozano-Soto et al., 2017). One study of tSMS over M1 suggested inhibitory effects on pinch force (Nakagawa and Nakazawa, 2018) while another found improved reaction times in an implicit motor learning task (Nojima et al., 2019). Studies support favorable safety and tolerability when tSMS was administered for up to 120 min (Oliviero et al., 2015). The safety, tolerability, and behavioral effects of tSMS have not been explored in children. However, a large volume of safety evidence comes from decades of MRI use where millions of patients have been exposed to much higher doses (1-8T) and durations (hours of exposure) of static magnetic fields (to much larger areas of tissue) with no significant adverse effects (van Osch and Webb, 2014). Furthermore, guidelines on safety of static magnetic field exposure conclude the evidence does not indicate the presence of serious health effects given acute exposure to up to 8T fields (International Commission on Non-Ionizing Radiation Protection, 2009).

Given its potential ease of application in young children and therapeutically relevant effects on M1 excitability, we aimed to evaluate whether tSMS could alter M1 excitability and motor learning in typically developing children. To the best of our knowledge, this is the first study of tSMS in a pediatric population. We conducted a randomized, sham-controlled, double-blinded, cross-over trial, hypothesizing that contralateral (right) M1 tSMS would decrease MEP amplitude and inhibit motor learning in the left hand.

## Materials and Methods

### Trial Design

The Pediatric Transcranial Static Magnetic Field Stimulation to Improve Motor Learning (PSTIM) trial was a randomized, double-blinded, sham-controlled, three-arm, cross-over interventional trial. Methods complied with the consolidated standards of reporting trials (CONSORT) guidelines including pediatric considerations (Schulz et al., 2010). The trial was registered with www.clinicaltrials.gov (NCT03949712). Methods were approved by the University of Calgary Research Ethics Board (REB 18-0178).

### Population

School-aged children were recruited through the population-based, volunteer Healthy Infants and Children Clinical Research Program (HICCUP^1^). Inclusion criteria were (a) written informed consent/assent, (b) age 8–18 years, (c) right-handed by self-report, and (d) typical development. Potential participants with any of the following were excluded: (a) diagnosis of any neurological, psychiatric or developmental disorder, (b) taking any neuroactive medications, (c) any contraindication to brain stimulation, and (d) pregnancy.

### Randomization, Blinding, and Concealment

Participants were computer-randomized into three groups which determined intervention order: (A) Sham, Right tSMS, Left tSMS; (B) Left tSMS, Sham, Right tSMS; and (C) Right tSMS, Left tSMS, Sham. A second randomization assigned the sham stimulation side, such that half of the participants in each group had sham on the left and half on the right. Participants, parents and the primary researcher conducting tSMS and analysis were blinded to randomizations. Only a research assistant was unblinded in order to apply the correct intervention. Participants were asked to guess if they received real or sham tSMS. The randomization code was broken to the primary researcher only after the final outcome and initial analysis was completed.

### Outcome Measures

#### Neurophysiological

The primary outcome of this study (and the main neurophysiological outcome) was right M1 excitability as measured by mean MEP amplitude generated in the left first dorsal interosseous (FDI) muscle. This was chosen to enable comparison of M1 excitability before and after application of the tSMS intervention. TMS is an established, safe and well tolerated method in children (Zewdie and Kirton, 2016). Previously described single and paired-pulse TMS methods were applied to assess motor system neurophysiology (Zewdie and Kirton, 2016). All experiments took place in the Alberta Children’s Hospital Pediatric Neurostimulation Laboratory where children had opportunity to test procedures beforehand and watch movies when possible for distraction from the TMS stimulation and to help reduce potential fatigue.

To measure MEPs, surface electromyography (EMG) was recorded by placing Ag/AgCl electrodes on the belly of the FDI muscle. A reference electrode was placed on the second phalange, with a ground on the ulnar head. EMG signals were amplified x1000 (2024F Isolated amplifier; Intronix Technologies Corp, ON, Canada), band-pass filtered (20–2000 Hertz (Hz)), and recorded (CED1401 signal analog/digital converter; Cambridge Electronic Design, Cambridge, United Kingdom).

First, single-pulse TMS (Magstim 200, Magstim, Cardiff, United Kingdom) used a flat iron Magstim TMS coil to locate the right and left M1 “hotspots”, defined as the location producing the largest and most consistent MEP (Zewdie and Kirton, 2016). The coil was placed at a 45-degree angle to the midline to induce a posterior-anterior current using monophasic waveforms. The identified “hotspot” was marked using neuronavigation (Brainsight2, Rogue Research, Montreal) to facilitate accurate coil replacement for serial measurements. Single-pulse TMS was then delivered to determine resting motor threshold (RMT) defined as the lowest stimulation intensity producing a 50 microvolts (μV) MEP in 5/10 stimulations. Ten suprathreshold (120% RMT) stimulations were administered to estimate cortical excitability.

Paired-pulse TMS was then completed using two connected stimulators (Magstim bi-stim, Magstim, Cardiff, United Kingdom). Consistent with other studies, pairs of pulses were delivered, which included a conditioning stimulus (CS) (80% RMT) followed by a test stimulus (TS) (120% RMT) (Zewdie and Kirton, 2016). Interstimulus intervals (ISIs) of 2 ms and 10 ms were used to evoke short-interval intracortical inhibition (SICI) and intracortical facilitation (ICF), respectively. A total of 30 pulses were administered in random order: 10 test single pulses, 10 paired pulses (2 ms ISI), and 10 paired pulses (10 ms ISI).

MEP signal files were imported into MATLAB R2011b (Mathworks, Inc., Natick, MA, United States) for offline analysis. Visual inspection was used to identify artifacts including baseline motor activity; proportion of traces removed was less than 5%. Peak-to-peak MEP amplitude values were calculated using a custom MATLAB script, which identified maximum and minimum MEP values within 15–80 ms after TMS. Mean peak-to-peak MEP amplitudes were averaged. SICI and ICF ratios were computed by dividing the average MEP amplitude of the conditioned responses into that of the test stimuli alone.

#### Behavioral

The main behavioral outcome was change in the Purdue Pegboard Test (PPT) left-hand score (PPT_L_). This enabled assessment of motor skill performance before, during and after application of the tSMS intervention. The PPT is a validated simple motor task that requires both gross and fine motor skills, described in detail in the cited reference (Gardner and Broman, 1979). The PPT produces consistent motor learning curves in school-aged children across multiple sessions (Tiffin and Asher, 1948; Gardner and Broman, 1979; Ciechanski and Kirton, 2017; Cole et al., 2018). The PPT consists of four tasks. For the PPT_L_, the participant used their left hand to move as many metal pegs into holes in the pegboard as fast as possible in 30 s. Following a 1-min break, the same task was performed with the right hand (PPT_R_). Following another 1-min break, the task was performed using both hands at the same time (PPT_LR_). Finally, 1-min was given to assemble a pin-washer-collar-washer structure using alternating hands for each metal piece (PPT_A_). All sections were repeated three times and averaged.

### Intervention

**FIGURE 1:**
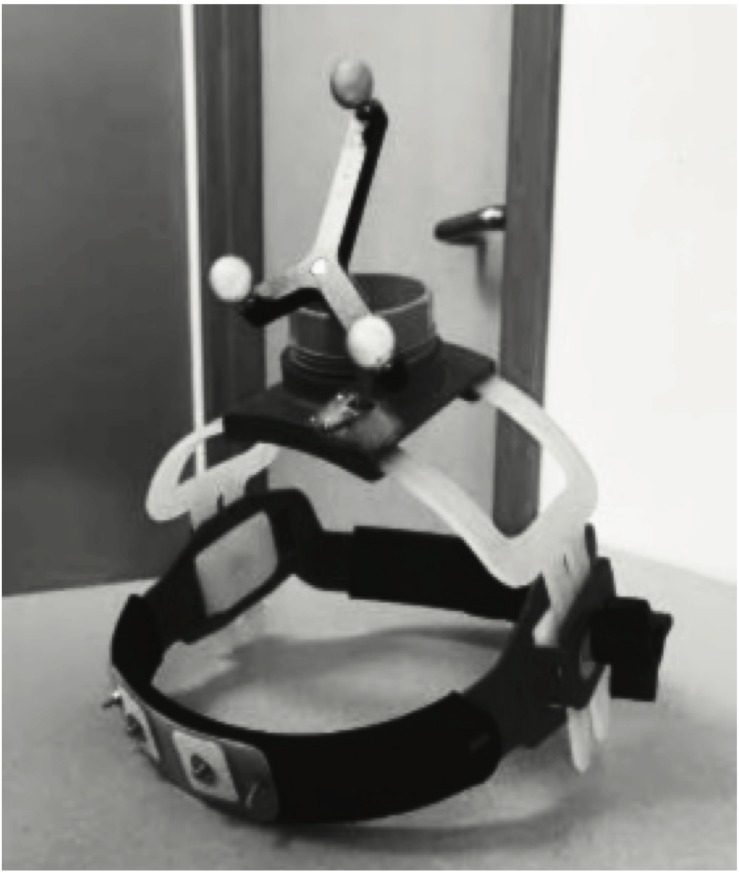
Custom-Engineered tSMS Helmet. Custom designed and partially 3-D printed helmet used for application of tSMS with neuronavigation.

The intervention was tSMS (or sham) over left M1 (ipsilateral) or right M1 (contralateral), modeled on previous adult studies (Oliviero et al., 2011; Silbert et al., 2013; Dileone et al., 2018). A strong cylindrical Neodynium magnet (S-45-30-N, Supermagnete) or a sham magnet (MAG45s, Neurek SL, Toledo, Spain) was affixed over the M1 hotspot using a custom-designed helmet (Figure 1). The sham magnet was identical in appearance and weight but carried no magnetic properties. The custom helmet allowed for movement in the anterior-posterior, superior-inferior and medial-lateral directions. Neuronavigation was utilized to place the magnet over the previously identified hotspot. The magnet was applied with South polarity (determined using a compass), consistent with previous adult studies (Oliviero et al., 2011). Magnet dimensions were 30 mm tall × 45 mm wide with an estimated strength of 300–450 milliTesla (mT) at the cortex (Tharayil et al., 2018).

### Study Flow

**FIGURE 2:**
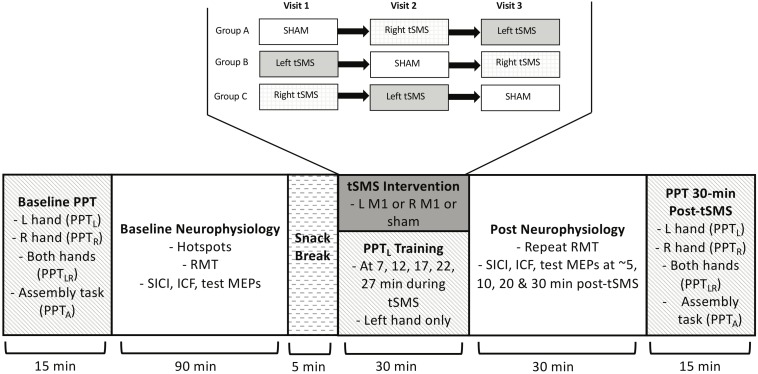
PSTIM protocol. Participants completed baseline PPT (all tasks: PPT_L_, PPT_R_, PPT_LR_, PPT_A_), and then underwent baseline neurophysiology testing, including determining the “hotspot”, identifying the RMT, and performing single and paired pulse protocols for test MEPs, SICI and ICF. They then received the tSMS intervention paired with motor training on the PPT_L_. There were 3 treatment orders, as shown. Neurophysiology measures (single and paired pulse) and PPT (all tasks) were repeated at multiple time intervals post-tSMS.

The timeline and flow of the study is diagrammed in Figure 2. On visit 1, all baseline behavioral and neurophysiological measures were obtained. Participants first performed the PPT_L_ outcome. Additional behavioral outcomes of PPT_R_, PPT_LR_ and PPT_A_ were then performed. The M1 hotspots for FDI were mapped followed by the single and paired pulse measurements, including RMT.

Following a short break, the magnet-holding device was affixed to the participant’s head. Size was adjusted for head shape and comfort. The magnet was positioned on the skull over the left or right M1 hotspot as identified by neuronavigation. The magnet was then held in place for 30 min. During this time, participants trained the non-dominant left hand by performing the PPT_L_ five times (minutes 7, 12, 17, 22, and 27). The non-dominant hand was targeted to enable skill growth from baseline, given the common assumption of lower skill in the non-dominant hand. TMS studies have also suggested differences in excitability between the dominant and non-dominant hemisphere (Daligadu et al., 2013) but we could only examine one. Use of the non-dominant hand is consistent with prior motor skill learning research by our team and others (Ciechanski and Kirton, 2017; Cole et al., 2018).

After completion of tSMS, the magnet and holding device were removed. The RMT of the right M1 hotspot (identified via neuronavigation) was then re-measured and the TMS neurophysiological measurements were repeated at minutes 5, 10, 20, and 30 post-tSMS. Finally, all PPT tasks (PPT_L_, PPT_R_, PPT_LR_, and PPT_A_) were repeated at approximately 35 min post-tSMS.

On visits 2 and 3, all procedures were repeated, with the exception of varying the intervention according to the randomized group. Each visit was scheduled to occur not less than two and not more than 4 weeks (+/- 4 days) from the previous one.

### Safety and Tolerability

Participants completed a pediatric tSMS and TMS safety and tolerability survey at the end of each session as previously described (Garvey and Gilbert, 2004; Cole et al., 2018). Participants were asked to rank the tolerability of the tSMS or TMS session in comparison to seven common childhood experiences (e.g. birthday party, shot at the doctor). Participants were also asked to report the presence and severity of any symptoms experienced including headaches, neck pain, unpleasant tingling or itching, fatigue, nausea, and light-headedness. All procedures were performed by trained personnel. Requests for additional breaks were accommodated.

### Sample Size

Sample size was determined based on the primary outcome (MEP amplitudes) using effect sizes in adults as a guide. Based on our crossover design, an expected (conservative) decrease of MEP amplitude in the stimulated M1 from approximately 1 millivolts (mV) to 0.9 mV compared to no change in sham, power of 90%, standard deviation (SD) of 0.1, and alpha of 0.05, we estimated a sample size of 24 (8 per group).

### Statistical Analysis

Given our primary neurophysiological and secondary behavioral outcomes, crossover design, and aim to explore effects both between and within subjects, we employed a linear mixed effects model. Fixed effects were considered for treatment (left tSMS, right tSMS or sham), visit (1, 2 or 3), age, and an interaction between treatment and visit (visit effects were only considered for PPT). For change in PPT_L_, PPT_R_, PPT_LR_, and PPT_A_ on visit 1 only, we also employed a simple linear regression with independent variables of treatment and age. The Shapiro–Wilks test was used to assess normality of distribution of residuals, and the Breusch-Pagan/Cook-Weisberg test was used to assess heteroskedasticity of residuals. Analyses of variance (ANOVAs) were also utilized to compare group demographics and ensure there were no significant differences in age, sex, and baseline PPT scores between groups. Analyses were performed using Stata 14.2.

## Results

### Population

A total of 131 potential participants were approached. Thirty participants were recruited. Six were subsequently excluded due to self-withdrawal for scheduling conflicts (*n* = 2), incorrect order of intervention (*n* = 1), and high RMT that precluded the TMS protocol (*n* = 3). The final sample of participants who consented and completed the study consisted of 24 participants (13 males) with a median age of 15.9 years (range 10–18). Participant demographics and baseline motor function are summarized in Table 1. Groups were comparable with no differences in age, sex, or function.

**TABLE 1:** Participant demographics and baseline PPT_L_ scores^1^.

	Group A	Group B	Group C	Mean
Age	14.96 (2.60)	15.12 (2.19)	15.63 (1.92)	15.23 (2.25)
Sex (F:M)	4:4	4:4	3:5	11:13
Baseline PPT_L_	14.17 (1.67)	13.67 (0.84)	14.04 (1.17)	13.96 (1.23)
Baseline PPT_R_	16.79 (0.80)	15.83 (1.46)	15.25 (1.93)	15.29 (1.55)
Baseline PPT_LR_	13.25 (1.23)	12.33 (0.78)	12.13 (0.89)	12.57 (1.07)
Baseline PPT_A_	36.21 (8.38)	37.08 (5.95)	35.42 (5.82)	36.24 (6.55)

*^1^Values reported are group means (SD), with the exception of sex, reported as a ratio of females:males.*

### Baseline Neurophysiology Measurements

**FIGURE 3:**
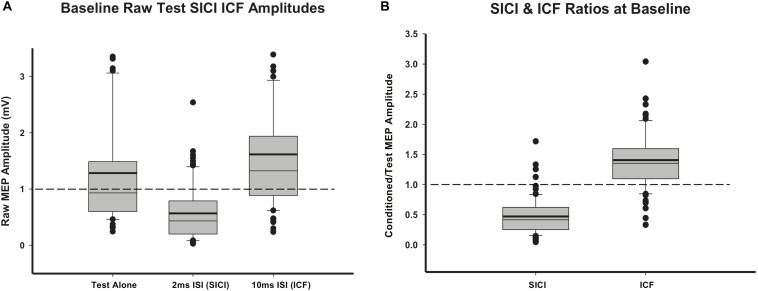
Baseline TMS data. **(A)** Baseline raw MEP values for test-pulse MEPs and paired-pulse (SICI and ICF) MEPs. **(B)** SICI and ICF ratios at baseline, calculated as raw SICI or ICF MEPs divided by raw test MEPs. Thick lines indicate mean value.

TMS data were obtained from all participants. RMT ranged from 37% to 77% of maximum stimulus output (MSO) (mean 49.29, SD = 9.61). Baseline RMT was negatively correlated with age (*r* = −0.56, *p* < 0.01). Mean (SD) test MEP amplitude from all participants at baseline was 1.28 (1.1) mV. SICI and ICF were present with test MEP inhibited by the 2 ms subthreshold CS (0.57 (0.5) mV) and facilitated by the 10 ms CS (1.62 (1.29) mV). Raw MEP averages are shown in Figure 3A. Mean ratios of raw conditioned/raw test MEPs for SICI (0.47 (0.31)) and raw conditioned/raw test MEPs for ICF (1.41 (0.48)) were robust and consistent with expected SICI and ICF ratios in children (Figure 3B).

### Effects of tSMS on M1 Neurophysiology

**FIGURE 4:**
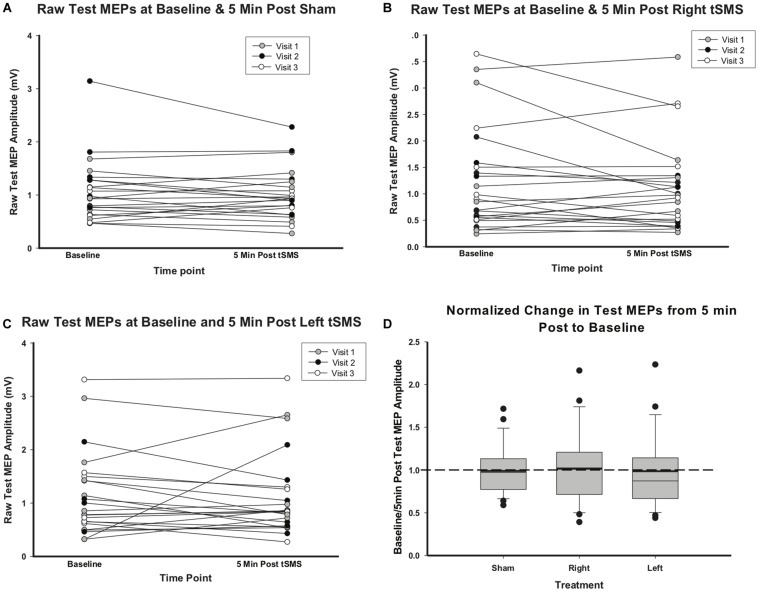
Effect of sham, left and right tSMS on corticospinal excitability. **(A)** Raw test MEP values at baseline and 5 min post-sham tSMS. **(B)** Raw test MEP values at baseline and 5 min post-right tSMS. **(C)** Raw test MEP values at baseline and 5 min post-left tSMS. **(D)** Change in test MEPs from baseline to 5 min post-tSMS, normalized to baseline test MEPs. Thick lines indicate mean value.

MEP amplitudes did not change significantly between baseline and the immediate (5 min) post-tSMS measurement regardless of treatment group. Change in individual raw test MEP amplitudes are shown in Figure 4 by treatment group. MEP amplitudes 5 min post-left or -right tSMS and normalized to baseline did not change as compared to sham (left tSMS 95% confidence interval (CI) −0.19, 0.64; *p* = 0.29; right tSMS 95% CI −0.38, 0.44; *p* = 0.89). In addition to these results for the presumed maximal effect time at 5 min, no changes were seen at 10, 20 or 30 min post-tSMS either. RMT also did not change between baseline and follow-up for any treatment group.

**FIGURE 5:**
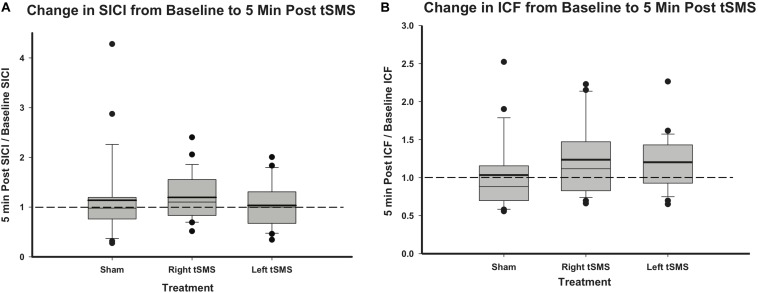
Effect of sham, right and left tSMS on intracortical excitability. **(A)** Change in SICI ratio from baseline to 5 min post-tSMS, calculated as SICI ratio 5 min post-tSMS divided by the SICI ratio at baseline. **(B)** Change in ICF ratio from baseline to 5 min post-tSMS, calculated as ICF ratio 5 min post-tSMS divided by the ICF ratio at baseline. Thick lines indicate mean value.

Measurements of SICI and ICF ratios from baseline to immediately post-tSMS are summarized in Figure 5. Changes in intracortical physiology following right tSMS, left tSMS and sham tSMS and normalized to baseline did not appear different between groups (all *p* > 0.24). Although results are only shown for 5-min post-tSMS, no significant changes were observed at 10, 20 or 30 min post-tSMS either (all *p* > 0.1).

### Effects of tSMS on Motor Learning Behavior

**FIGURE 6:**
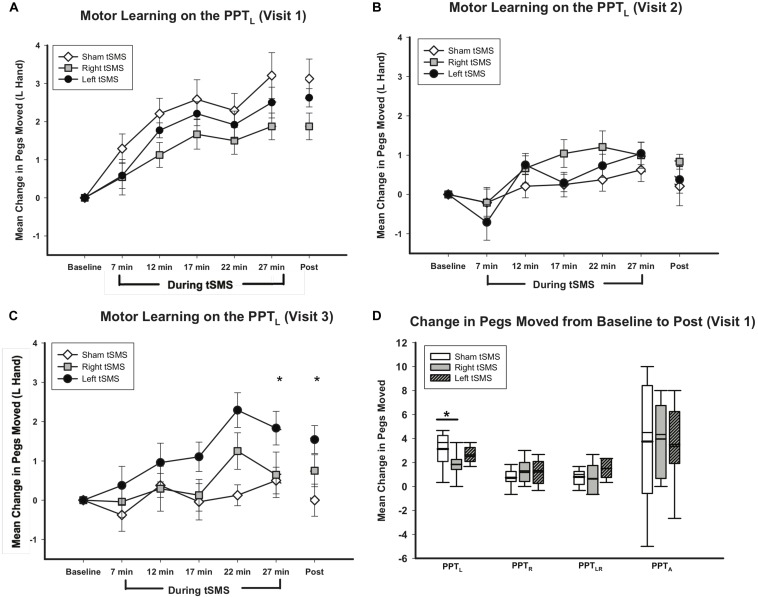
Trained left hand motor learning by intervention. **(A–C)** Show trained left hand motor learning on PPT_L_, **(D)** shows change in all PPT tasks. **(A)** Mean change in pegs moved from visit 1 baseline for PPT_L_ was greater for sham (white diamonds) than for left (gray squares) or right tSMS (black circles). Effects were retained for all groups 30 min post-tSMS. Error bars show standard error. *p < 0.01 for right tSMS vs sham. **(B)** Mean change in pegs moved from visit 2 baseline for PPT_L_ were not statistically significantly different for sham (white diamonds), left (gray squares) or right tSMS (black circles) on visit 2. **(C)** Mean change in pegs moved from visit 3 baseline for PPT_L_ was greater for left (black circles) than for sham (white diamonds). Effects were retained 30 min post-tSMS. Error bars show standard error. *p < 0.01 for left tSMS vs sham. **(D)** Mean change in pegs moved from baseline for PPT_L_ was greater for sham (white box) than for left (striped box) or right tSMS (gray box) at 30 min post tSMS. No statistically significant changes in pegs moved occurred for PPT_R_, PPT_LR_, or PPT_A_ for left or right tSMS compared to sham. Thick lines indicate mean value. Error bars show standard error. *p < 0.01 for right tSMS vs sham for PPT_L_ on visit 1.

All participants demonstrated motor learning curves consistent with previous pediatric studies (Ciechanski and Kirton, 2017; Cole et al., 2018). Curves of motor learning by intervention for visit 1 are shown in Figure 6A. Learning differed by intervention group. On average, participants who received sham tSMS improved by 3.21(1.70) pegs by their final PPT. Those receiving left M1 tSMS improved by 2.50(1.14) pegs by their final PPT. Participants receiving right M1 tSMS improved by 1.88(0.99) pegs on their final PPT. The linear mixed effects model conditional upon age and visit suggested the effect of left tSMS compared to sham was a 0.74 *reduction* in pegs moved (95% CI −1.72, 0.25;*p* = 0.14). The effect of receiving right tSMS compared to sham was a 1.47 *reduction* in pegs moved (95% CI −2.46, −0.48;*p* < 0.01). The Cohen’s *d* for sham versus right tSMS was 0.96. The same pattern of group differences was observed at the retention timepoint 30 min following completion of tSMS with a 1.36 *reduction* for right tSMS (95% CI −2.33, −0.39; *p* < 0.01) and 0.52 *reduction* (95% CI −1.48,0.45; *p* = 0.26) for left tSMS. No significant treatment group effects were seen for the other motor outcomes (Figure 6D). Greater variance in these other secondary motor outcomes is consistent with other similar studies. The Shapiro–Wilks test and the Breusch-Pagan/Cook-Weisberg test revealed normality and homoskedasticity of residuals could not be rejected, giving us confidence in our estimation methodology.

**FIGURE 7:**
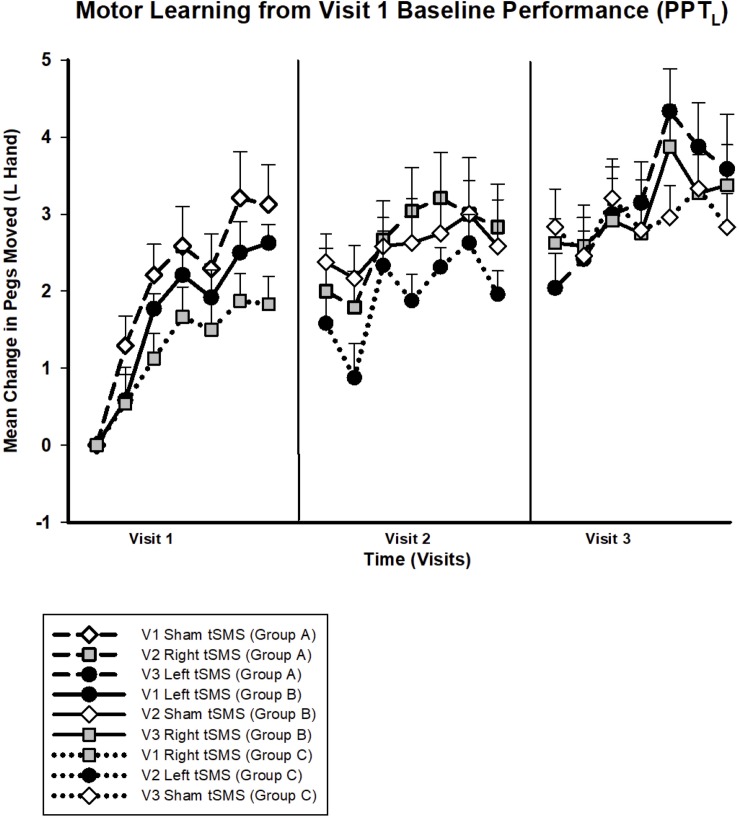
Trained left hand motor learning by intervention for all visits. Mean change in pegs moved from visit 1 baseline for PPT_L_. All groups experienced motor learning from baseline. Symbols indicate intervention: sham (white diamonds), right (gray squares) or left tSMS (black circles). Lines indicate participant group: group A (dashed), group B (straight), group C (dotted), for which each group contains the same participants. Error bars show standard error.

No significant changes were seen in the PPT_L_ task on visit 2 (Figure 6B) or any other secondary behavioral outcomes. On visit 3, treatment group specific differences were observed in motor learning curves (Figure 6C). Participants who received left tSMS experienced a *greater* improvement in PPT_L_ scores compared to the sham group. Conditional on age and visit, those who received left tSMS moved 1.47 *more* pegs than those receiving sham (95% CI −0.48, −2.46; *p* < 0.01). The Cohen’s *d* for sham versus left tSMS was −1.22. The improvement with left tSMS compared to sham was consistent at the retention timepoint 30 min following tSMS (1.60 improvement, 95% CI 0.64, 2.58; *p* = 0.001). Change in pegs moved for those who received right tSMS did not differ from the other groups (*p* = 0.62). The sham group did not change from baseline on visit 3. Motor learning from original baseline for all participants by treatment group is shown in Figure 7.

### Tolerability and Safety of tSMS

**FIGURE 8:**
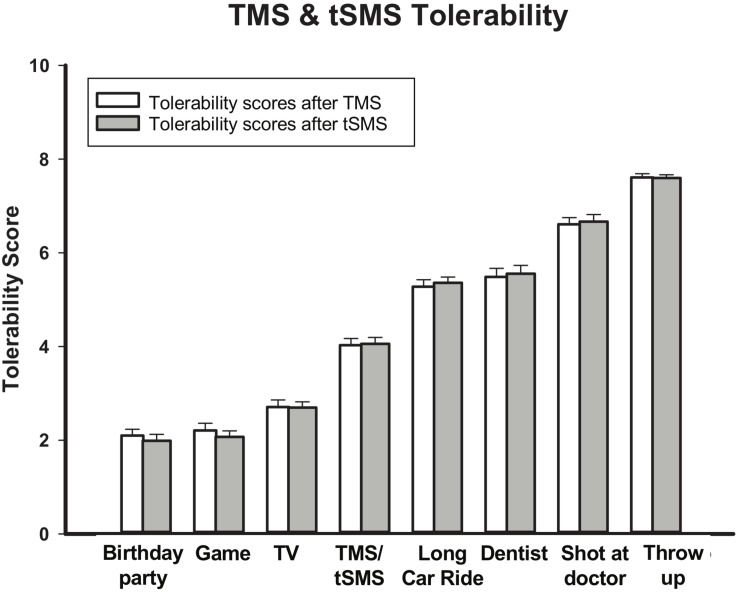
tSMS and TMS Tolerability. Bars show tolerability score out of 8 (higher scores indicate less tolerability for various childhood experiences in comparison to TMS and tSMS. tSMS and TMS are both more tolerable than a long car ride, but less tolerable than watching TV. Error bars show standard error.

A total of 72 tSMS sessions were completed without any serious adverse events. The most common reported side effects of tSMS were headaches (Real: 19% mild, 2% moderate; Sham: 25% mild) and neck-pain (Real: 19% mild, 2% moderate; Sham: 8% mild). Other reported side effects were fatigue (Real: 8% mild, 2% moderate; Sham: 8% mild), light-headedness (Real: 2% mild; Sham 8% mild) and unpleasant tingling (Real: 2% mild; Sham 4% mild). On the pediatric brain stimulation tolerability scale, mean tSMS score was 4.06/10 (+/−1.17). This average ranked as less favorable than watching television (TV) (2.70) but more favorable than a long car ride (5.36) (Figure 8). The 144 TMS neurophysiology sessions were also well tolerated. The most common side effects were fatigue (24% total; 21% mild, 3% moderate) and headaches (14% total; 13% mild, 1% moderate). Others were neck pain (11% mild), unpleasant tingling (4% mild) and light-headedness (3% mild). Mean tolerability score was 4.03 (+/- 1.21), again falling between watching TV (2.07) and a long car ride (5.28) (Figure 8). When participants were asked if they would recommend the study to a friend, 100% said yes (*n* = 21; three participants were missed).

## Discussion

In this trial, we evaluated the effects of tSMS on cortical neurophysiology and motor learning in a pediatric population. We show that tSMS is feasible, well tolerated and safe in school-aged children. Our results suggest that contralateral tSMS may have inhibitory effects on motor learning while stimulation of the ipsilateral hemisphere may enhance later stages of learning, although this requires additional study. We were not able to replicate the neurophysiological effects of tSMS reported in most adult studies.

Since the introduction of tSMS (Oliviero et al., 2011), numerous studies have tested the effect of contralateral tSMS on cortical neurophysiology over M1 and other cortical brain regions in healthy adults. The most consistent net tSMS effects have been inhibitory in nature, often demonstrating reduced excitability such as in the motor cortex where TMS-evoked MEP amplitudes are reduced (Oliviero et al., 2011; Silbert et al., 2013; Dileone et al., 2018). The mechanism behind tSMS is not yet known, but it is thought that tSMS may act by indirectly altering ion channels in cell membranes (Rosen, 2003). Surprisingly, contralateral tSMS in children did not generate similar results. Although we hypothesized there would be an inhibition of MEP amplitudes, we found no evidence of consistent effects of contralateral (or ipsilateral) tSMS on any of our neurophysiological outcomes. This included our measures of intracortical motor neurophysiology (SICI and ICF) where again no effects were observed.

Multiple potential contributing factors may account for this discrepancy from the tSMS effects described in adults. Our study tested tSMS for the first time in a pediatric population where the many known differences of the developing brain may have been a factor. Many other studies of different forms of NIBS (TMS, tDCS) have identified distinct differences in effects in children as compared to adults (Moliadze et al., 2015; Ciechanski et al., 2018). It may also be more difficult to discern changes in intracortical neurophysiology in children compared to adults. For example, SICI may be more difficult to elicit in children, and can be differentially affected by practice- or use-dependent plasticity (Garvey and Mall, 2008). The combination in our study of both a pediatric population and concomitant motor training and neuromodulation by tSMS may have further complicated our ability to detect changes in TMS measures of M1 neurophysiology.

TMS data are also intrinsically noisy. TMS neurophysiology outcomes depend on a variety of factors, such as muscle contraction, fatigue, and attention (Darling et al., 2006; Li et al., 2015). Additional factors influencing TMS neurophysiology established in adults such as gender, sleep, medications, and genetics have not been well defined in children (Ridding and Ziemann, 2010). Reliability of the measures themselves also varies. Studies of test-retest reliability in adults have established the natural variability in the data and also that this variability changes for different measures. For example, reliability of ICF and SICI measures are lower than for RMTs (Schambra et al., 2015; Hermsen et al., 2016). Even simple test stimuli demonstrate greater variability when participants are relaxed as compared to holding an active contraction (Darling et al., 2006). While the same reliability studies have not been completed in children, there are reasons to expect the same issues are at least as relevant, if not potentially more so.

Another factor unique to our study design was that TMS measures of neurophysiology had to be acquired in conjunction with the execution and measurement of motor training. This not only complicates the measurements themselves but introduces potential noise from the effects of motor learning. Previous literature assessing motor learning has shown effects on cortical neurophysiology. For example, multiple studies have shown increases in MEP amplitudes measured from hand muscles following hand motor training (Muellbacher et al., 2001; Cirillo et al., 2010). Furthermore, pharmacological studies have shown that plastic changes associated with motor learning may share mechanistic similarities with neurostimulation such as long-term potentiation (Butefisch et al., 2000). It is therefore possible that our pairing of motor training with tSMS may have altered the potential neurophysiological effects of tSMS (Foffani and Dileone, 2017). Further studies comparing tSMS alone versus tSMS combined with motor training in children would be required to determine this.

Behaviorally, we were able to demonstrate that tSMS over M1 may modulate motor learning in children. Effects appeared to be specific to both the side of stimulation and timing across multiple motor learning sessions. Consistent with our clinical hypothesis, right contralateral M1 tSMS significantly inhibited motor learning in the trained, left hand as assessed by the PPT_L_. Effects were consistent across the learning curve which itself was comparable to previously described single day PPT learning curves in children (Ciechanski and Kirton, 2017; Cole et al., 2018). Specificity of effect to the trained hand was further suggested by the absence of other significant changes in any of the other secondary motor function outcomes (PPT_R_, PPT_LR_, PPT_A_). While our observed behavioral effects require replication, they would appear to be of similar magnitude and effect size as described for more studied forms of M1 non-invasive neuromodulation.

In contrast to the inhibitory effects of contralateral (right) tSMS on left hand motor learning, we found that after multiple days of motor training, ipsilateral (left) tSMS had a facilitatory effect on left hand motor learning as measured by the PPT_L_. This potential effect was hypothesized *a priori* based on previous studies of the effects of ipsilateral M1 rTMS and tDCS on hand motor learning. Though many exceptions are now recognized, anodal tDCS has often been suggested to increase cortical excitability while cathodal-tDCS may decrease cortical excitability (Batsikadze et al., 2013; Monte-Silva et al., 2013). In keeping with this simple model, previous adult studies have shown that anodal tDCS can facilitate motor learning when applied to the contralateral M1 (Vines et al., 2006; Reis and Fritsch, 2011). In addition, cathodal tDCS applied to the opposite, ipsilateral M1 has also been shown to facilitate motor learning (Reis and Fritsch, 2011). One study comparing these effects of M1 tDCS on motor learning directly (Vines et al., 2006) found that cathodal tDCS applied ipsilaterally improved motor learning, contralateral cathodal tDCS inhibited it, and anodal tDCS had the opposite effects (contralateral improvement, and ipsilateral inhibition). This body of adult evidence supports the concept that cathodal tDCS may improve motor learning via modulation of well-established inhibitory transcallosal pathways (interhemispheric inhibition (IHI)) which itself is associated with motor function in adults (Williams et al., 2010). Such “disinhibition” by ipsilateral cathodal stimulation might enable relative “excitation” of the opposite motor cortex, in turn facilitating motor learning (Vines et al., 2006). That TMS measures of IHI appear to be similar in school-aged children as compared to adults (Ciechanski et al., 2017) further supports this premise.

Translationally, these behavioral effects of M1 tSMS may be relevant to stroke rehabilitation. A theory of IHI imbalance has dominated early approaches to non-invasive neuromodulation of the contralesional hemisphere, though this model has more recently been questioned. Neuromodulation strategies aiming to reduce cortical excitability in the contralesional hemisphere have been associated with improved motor performance in chronic stroke (Hsu et al., 2012; Elsner et al., 2017).

Although the underlying models are different, a smaller but significant body of evidence has supported the same approach of inhibiting the contralesional motor cortex in children with PS and HCP (Kirton, 2013b). Substantial preclinical (Martin et al., 2007; Friel et al., 2013; Friel et al., 2014; Wen et al., 2018) and human (Eyre, 2007; Staudt, 2007b) evidence following early brain injury supports a negative association between the relative preservation of ipsilateral corticospinal projections from the contralesional hemisphere to the affected hand and clinical motor function. Targeting these ipsilateral tracts on the contralesional side thus has the potential to improve motor function. Multiple translational trials show both contralesional low frequency rTMS (Gillick et al., 2014; Kirton et al., 2016) and cathodal tDCS (Kirton et al., 2017; Gillick et al., 2018) may enhance therapy-induced gains in clinical function.

Our results here provide preliminary evidence that tSMS might provide an alternative application to achieve similar M1 inhibitory effects, although additional research exploring this is needed. The simplicity of tSMS, including potential ease of application to very young children, possibly paired with infant therapy in the home environment, is particularly appealing.

Our study has also established the tolerability and feasibility of tSMS in children. Rankings of tolerability were comparable with pediatric studies of motor cortex TMS and tDCS (Kirton et al., 2016; Cole et al., 2018; Zewdie et al., 2018). The most common side effects were mild headaches and neck pain. By both our observations and subject report, we believe many of these effects were largely due to the weight of the magnet itself. While our tSMS magnet weighed only 360 g (less than 1 pound), its mass was relatively highly concentrated on a small area of the skull due to its small diameter. Additional modifications to either better support the weight of the magnet (or otherwise redistribute the weight) may be helpful in improving tolerability further.

Several important limitations are noted. Our study was limited by an informed but modest sample size of 24 participants. Given the variability of TMS outcomes discussed above, larger sample sizes would certainly have been beneficial. Furthermore, with only 8 participants per treatment group, our ability to detect specific differences may have been reduced, emphasizing the need for our crossover design to be replicated in future studies. The crossover design did increase power for our neurophysiological outcomes but posed challenges for our clinical outcomes for which there may have been carry-over effects. Motor training on the PPT does not fully wash-out and can reach a plateau. As such, effect sizes on visits two and three were limited by previous motor training, and potentially by the intervention(s) received on prior visits. Therefore, potential motor learning effects of tSMS require additional studies designed primarily to assess behavioral outcomes. The 3-h study visits were tiring, especially for younger participants, although we tried to mitigate this with a snack break midway through each visit. We were not able to account for all factors that might have influenced our outcomes including fatigue, genetics, and attention (Li et al., 2015).

Ultimately, our data suggests that tSMS over M1 may modulate motor learning in children with specific effects of location and timing but this finding would benefit from further research. Our results also suggest that neurophysiological changes may differ in children compared to adults, and further research to determine neurophysiological effects of tSMS is required. Translationally, this study opens new opportunities for exploration into clinical trials of tSMS as a simple, non-invasive method to modulate motor learning in children with CP, with the ultimate goal of home-based, personalized, neuromodulation therapy during optimal windows of developmental plasticity.

## Data Availability Statement

All relevant data generated and analyzed for this study is included in the article.

## Ethics Statement

The studies involving human participants were reviewed and approved by the University of Calgary Research Ethics Board. Written informed consent to participate in this study was provided by the participants or participants’ legal guardian/next of kin.

## Author Contributions

AHo contributed to the study design, recruitment of participants, data collection and analysis, and drafting and revising the manuscript. EZ contributed to the study design, data collection, data analysis, and revising the manuscript. HC, AHi, and H-CK contributed to the data collection, drafting and revising the manuscript. AN-A contributed to the data analysis and revising the manuscript. AK contributed to the obtaining funding, study design, and revising the manuscript. All authors reviewed and approved the final version for submission.

## Conflict of Interest

The authors declare that the research was conducted in the absence of any commercial or financial relationships that could be construed as a potential conflict of interest.

## Footnotes

1 www.hiccupkids.ca
